# Validation of a Locally Developed Life Skills Development Package (the Manohari Module) for Adolescents in Sri Lanka: Evidence From Expert Review and Adolescent Feedback

**DOI:** 10.7759/cureus.113282

**Published:** 2026-07-24

**Authors:** Pethirupillai AD Coonghe, Mahesan Ganeshan, Rajendra Surenthirakumaran, Cyril J Mathanraj, Nadarajah Navaneetham, Grace Hensman, Kamshana Kajendran

**Affiliations:** 1 Department of Community and Family Medicine, Faculty of Medicine, University of Jaffna, Jaffna, LKA; 2 Department of Psychiatry, Ministry of Health, Colombo, LKA; 3 Department of Public Health, Ministry of Health, Colombo, LKA; 4 Department of Marketing, University of Jaffna, Jaffna, LKA; 5 Department of Pharmacy, Faculty of Allied Health Sciences, University of Jaffna, Jaffna, LKA

**Keywords:** adolescents, child health, judgmental validation, life skills, manohari, mental health, qualitative research, sri lanka

## Abstract

Background

Adolescent mental health is a growing public health concern, particularly in low- and middle-income countries where access to mental health services is limited. Life skills-based interventions have been identified as effective strategies for promoting mental well-being. This study aimed to validate the locally developed life skill developmental package (the Manohari module) using a mixed-methods approach to support its implementation among adolescents.

Methods

A mixed-methods approach was employed. Expert validation was conducted using the modified Delphi method with a panel of six experts, including two psychiatrists, two community physicians, and two Zonal Directors of Education (ZDEs). The panel evaluated the module's content validity, cultural appropriateness, and clinical relevance. Perspectives from the ZDEs were obtained to evaluate feasibility and integration within the school system. A structured evaluation form, adapted from the literature, was used, comprising eight core items rated on a five-point Likert scale, along with subject-specific items and open-ended questions, to gather qualitative feedback. Additionally, a focus group discussion (FGD) was conducted with six Grade 12 students to explore acceptability, understanding, and perceived usefulness. Expert ratings for each assessment item were summarized using percentage agreement. Items with less than 65% agreement were identified for further review based on expert feedback. Qualitative data from the FGD were analyzed using thematic analysis. Ethical approval was obtained from the Ethics Review Committee, Faculty of Medicine, University of Jaffna.

Results

Overall, the module evaluation gave positive responses, with most expert responses falling within the "agree" and "strongly agree" categories; five of the eight core items achieved over 65% agreement. The majority of experts agreed that the module content was relevant, culturally appropriate, and practical for delivery by non-health professionals. However, variability in agreement was observed regarding clinical relevance and developmental appropriateness. Qualitative feedback highlighted the need for improved developmental targeting and inclusion of context-specific adolescent challenges. ZDEs indicated strong agreement for integration within the school system, although further contextual adaptation was recommended. Findings from the FGD demonstrated high acceptability, with participants reporting the module was engaging, relatable, and easy to understand. However, the perceived impact on behavior change was moderate, suggesting the need for stronger motivational and interactive elements.

Conclusion

This study provides preliminary evidence supporting the acceptability, content validity, and cultural relevance of the Manohari module as a life skills development package for adolescents in Sri Lanka. Experts' and adolescents' feedback highlighted areas for improvement, particularly developmental appropriateness and contextual adaptation, which will guide future refinement of the module. Further validation with larger and more diverse populations is needed before wider implementation, along with research evaluating the module's impact on adolescent mental health outcomes to support its integration into educational and community settings.

## Introduction

Adolescents represent a vital and dynamic segment of the global population, shaping the future health, social, and economic landscape of societies [1,2]. According to the World Health Organization (WHO), adolescents are defined as individuals aged 10-19 years, and there are approximately 1.3 billion adolescents worldwide, accounting for nearly 16% of the total population [3,4]. Adolescence is a transitional phase between childhood and adulthood, marked by neurobiological, physiological, psychological, and social development, during which individuals are more likely to engage in risk-taking behaviors [5].

Adolescent mental health is a critical and growing public health concern worldwide. It is estimated that one in seven individuals aged 10-19 years experiences a mental disorder, contributing to approximately 15% of the global burden of disease within this age group. Among adolescents, depression, anxiety, and behavioral disorders are leading causes of illness and disability [6]. Alarmingly, suicide ranks as the third leading cause of death among individuals aged 15-29 years, underscoring the severity of unmet mental health needs during this transitional life stage [7]. Adolescents living with mental health conditions are particularly vulnerable to a range of adverse outcomes, including social exclusion, stigma, reduced help-seeking behavior, educational challenges, engagement in risk-taking behaviors, poor physical health, and exposure to human rights violations [6]. Access to mental health services also remains particularly limited in low- and middle-income countries, where health systems often face constraints in resources, infrastructure, and trained personnel [8,9]. These multifaceted impacts highlight the urgent need for comprehensive, accessible, and youth-sensitive mental health interventions.

Sri Lanka has a population of approximately 22 million people, of which nearly one-fifth comprises adolescents [10,11], highlighting the significant demographic importance of this age group. The mental health of adolescents in Sri Lanka has been affected by the long-standing armed conflict, the Easter Sunday attacks, the COVID-19 pandemic, and the foreign currency crisis, along with the associated disruptions to schooling, increased screen time, and changes in daily life [12]. Evidence from Sri Lanka indicates that mental health problems among school-going adolescents are increasingly prevalent, yet remain insufficiently explored in terms of their determinants and contextual influences [10,13]. Studies indicate that adolescents in Sri Lanka are exposed to a range of mental health-related challenges, including food insecurity, involvement in physical fights, exposure to bullying, violence victimization, not living with both parents, overprotective paternal influence, increased academic stress, and daily social media use, all of which are associated with an increased risk of mental health and psychological problems [10,11]. Additionally, family dynamics and changing community structures play a crucial role in shaping adolescent mental health outcomes in the Sri Lankan context [14].

Life skills education has been widely recognized as an effective strategy for promoting mental well-being and preventing risk behaviors among adolescents. According to the WHO, life skills are defined as adaptive and positive abilities that enable individuals to effectively cope with the demands and challenges of everyday life [15]. Previous studies in Sri Lanka have demonstrated that structured educational and psychosocial interventions can significantly improve adolescents' well-being and coping abilities [13,16]. Despite these efforts, there remains a notable gap in locally developed, comprehensive, culturally appropriate life skills development packages tailored to the needs of Sri Lankan adolescents.

In response to emerging community-level mental health challenges, a peace-building initiative was undertaken, within which a community-based Mental Health and Psychosocial Support (MHPSS) program, titled "Manohari," was developed by a multidisciplinary team, with significant contributions from Dr. M. Ganesan and support from the WHO through the World Peace Fund.

The Manohari module has been utilized in various parts of the country since 2017 and has gained acceptance among diverse community groups, including school students, teachers, village communities, district and divisional secretariats, and mothers' clubs [17]. While anecdotal, non-scientific evidence suggests its usefulness, the module has not yet undergone formal scientific evaluation. Evaluating the content validity, cultural relevance, and contextual appropriateness of such interventions prior to wider implementation is essential to ensure their sustainability and effectiveness. Judgmental validation, which incorporates expert opinion and end-user perspectives, provides a systematic approach to determining whether an intervention aligns with the intended sociocultural and practical context [18,19]. Therefore, this study aims to judgmentally validate the Manohari module using a mixed-methods approach to support its implementation among adolescents.

## Materials and methods

A qualitative mixed-method approach was undertaken in this study to assess the judgmental validity of the Manohari module. The judgmental validity was assessed using the modified Delphi method [20] with subject experts to achieve consensus on the module content and using focus group discussions (FGDs) [21] conducted with a group of students to explore their perceptions of and acceptability of the module.

A purposive sample of six experts was recruited to participate in the modified Delphi process for content validation, while six students were recruited for an FGD to assess the module's clarity, acceptability, and cultural relevance. As this was an initial qualitative validation study, the sample size was considered adequate for obtaining in-depth feedback; however, the relatively small sample size may limit the generalizability of the findings and should be considered when interpreting the results.

Development of Manohari modules

Manohari is a community-based MHPSS programme developed collaboratively by the Directorate of Mental Health, Ministry of Health, Sri Lanka, and the WHO. The term "Manohari," derived from Sanskrit, denotes "stealing the heart or mind" (captivating), reflecting the programme's focus on engaging and positively influencing the hearts and minds of individuals to enhance mental well-being within the community.

The programme was designed to address the root causes of psychosocial distress and conflict at the community level through ten structured modules, which were developed by a multidisciplinary resource team of experienced MHPSS professionals [22]. These modules are structured as a life skills-based developmental package, incorporating interactive and activity-based sessions that address key domains relevant to psychosocial development. These include communication skills, emotional intelligence, problem-solving, decision-making, time management, stress management, and interpersonal relationships, aiming to enhance adaptive coping and overall mental well-being.

Initially, 10 drama therapy-based training modules were developed in collaboration with the WHO, with an additional four modules later incorporated, comprising activities such as storytelling, dialogues, role-playing, and interactive discussions focused on emotional regulation, coping skills, positive behavioral change, and improving understanding and non-violent responses in stressful situations. The modules are based on Panchatantra stories, Jataka tales, or similar scripts using animals as characters. The use of animal characters provides symbolic representations of human behaviors and emotions, enabling adolescents to discuss values, relationships, and coping strategies with reduced personal defensiveness while maintaining engagement through familiar cultural narratives. The facilitator presents the story to the participants and then encourages them to act it out. The programme consists of several topics, such as understanding emotions, rage, taking responsibility for one's feelings and changing behaviors, shyness, envy, peer pressure, keeping children safe and happy, recognizing the emotions of others and responding to them, and communicating needs effectively. This module is feasible for delivery in low-resource settings and can be implemented by non-mental health professionals. The programme was piloted across multiple districts in Sri Lanka, including Mullaitivu, Kilinochchi, Mannar, Vavuniya, Monaragala, Badulla, Gampaha, Hambantota, Kalutara, Kandy, Matara, and Ratnapura, targeting frontline mental health staff, MHPSS workers, and community leaders [17].

Experts review using the modified Delphi approach

An expert review using a modified Delphi approach, derived from the original method developed by Dalkey and Helmer [20], was used to evaluate the validity of the module. This method was used to systematically gather and synthesize expert opinions in a structured manner to facilitate consensus.

Experts were selected based on the following criteria to ensure their relevance and suitability for the study. Experts were required to: (i) hold recognized professional qualifications in their respective fields (psychiatry, community medicine, public health, or educational administration); (ii) have a minimum of five years of professional experience in their respective fields in Sri Lanka; (iii) demonstrate active involvement in public health and adolescent health programmes; and (iv) have prior experience in mental health programme development.

The panel of experts included two Zonal Directors of Education (ZDEs), two Consultant Psychiatrists, and two Consultant Community Physicians.

The expert panel reviewed the Manohari module to assess face and content validity, along with overall consensus. During the initial review, each expert was contacted via email and provided with information outlining the study and its objectives, along with the Manohari module. Experts were requested to assess the clarity and relevance of the module content.

A structured evaluation form, developed based on the literature [23,24], was used for this purpose. The form consisted of eight core items applicable to all the experts, rated on a five-point Likert scale (strongly agree, agree, neutral, disagree, and strongly disagree) to assess agreement and relevance. In addition, subject-specific items were included according to the expertise of the panel members. ZDEs received six additional subject-specific items. Consultant psychiatrists and community physicians received three common subject-specific items; consultant psychiatrists were given three further subject-specific items, while Community Physicians received one additional item. ZDEs and consultant psychiatrists each received 14 evaluation items, while Community Physicians received 12 evaluation items for the judgmental validation of the module. The evaluation form also included open-ended questions to obtain qualitative feedback and suggestions for improvement.

Following the initial round, responses were compiled and summarized, and the anonymized feedback was shared with the expert panel. Experts were then encouraged to review the summary and refine their opinions through continued email communication until consensus on the module's validity was achieved. As no further rating rounds or predefined consensus criteria were applied, this study represents an expert review using a modified Delphi approach rather than a formal Delphi validation.

Ethical approval for the study was obtained from the Ethics Review Committee, Faculty of Medicine, University of Jaffna (approval no. J/ERC/24/160/NDR/0320).

Data analysis involved deriving the percentage of agreement for each item, defined as the proportion of experts selecting either "strongly agree" or "agree." Items with less than 65% agreement were subjected to further review and revision based on the experts' feedback.

Student FGDs

In addition to expert feedback, students' perspectives were obtained to assess the face and content validity of the module. Six Grade 12 students were recruited from a secondary school outside Jaffna using convenience sampling. Following approval from the school administration, eligible students were invited to participate voluntarily in the study. To ensure equal representation, participants were selected to maintain an equal male-to-female ratio (1:1). The FGD, with a duration of approximately two hours, was facilitated by the principal investigator of the study. The FGD explored students' overall impressions of the module across several key dimensions, including the clarity of language, the reliability of characters and situations, cultural appropriateness, the portrayal of mental health, understanding of life skills, usefulness for real-life situations, and the quality of illustrations. Students were encouraged to share their perspectives openly.

The qualitative data from the FGD were analyzed using thematic analysis. The discussion notes were read several times to achieve familiarity with the data. Meaningful statements related to participants' perceptions of the module were identified and coded. Similar codes were then grouped into categories, from which overarching themes were developed through an iterative review process. The identified themes represented participants' views on the strengths of the module and areas requiring improvement.

The findings from the FGDs were used to further refine the module. By combining feedback from both the expert panel and the student participants, the module was comprehensively reviewed and improved to ensure clarity, relevance, and alignment with the intended objectives of the life skills training programme.

## Results

Overall expert agreement on the content of the Manohari module

For the purpose of content validation, experts were defined as professionals with specialized knowledge and practical experience relevant to the module content, comprising two consultant community physicians, two consultant psychiatrists, and two ZDEs.

The overall agreement regarding the module content is presented in Table 1. The findings indicate a generally positive evaluation by the expert panel, with most responses falling within the "agree" and "strongly agree" categories, as five out of eight core items achieved an overall agreement above 65%.

**Table 1:** Overall agreement on the content of the Manohari module as assessed by experts (n = 6)

No.	Assessment items (statements)	Agreement - n (%)
1	The topics covered are relevant to the adolescent population. (Statement_1)	4 (66.67)
2	The module aligns with the current understanding of adolescent mental health in Sri Lanka. (Statement_2)	4 (66.67)
3	The learning activities are practical and easily understandable for non-health professionals delivering the module. (Statement_3)	4 (66.67)
4	The module adequately covers key life skills needed for mental health promotion. (Statement_4)	5 (83.33)
5	The examples, scenarios, and language used are culturally sensitive and appropriate for Sri Lankan adolescents. (Statement_5)	3 (50)
6	The module considers the diverse cultural, religious, and socioeconomic backgrounds of adolescents in Sri Lanka. (Statement_6)	4 (66.67)
7	The activities and exercises are engaging and developmentally appropriate. (Statement_7)	3 (50)
8	The module addresses the pressure from academic expectations and familial expectations. (Statement_8)	3 (50)

A majority of the experts (83.33%) agreed that the module adequately covers key life skills needed for mental health promotion. Four experts (66.67%) agreed that the topics covered in the module are related to the adolescent population and align with the current understanding of adolescent mental health in Sri Lanka. However, qualitative feedback from the consultant psychiatrist indicated that only some topics are more applicable to late childhood and early adolescence, with limited relevance to late adolescence, and that the overall coverage of adolescent mental health issues remains insufficient.

Similarly, four experts (66.67%) also agreed that the learning activities are practical and easily understandable for non-health professionals delivering the module. The consultant psychiatrist noted that while the activities are highly practical for grassroots or community-level workers, the level of comprehension may vary. The consultant community physician emphasized the need for additional simplification of the learning activities.

Regarding cultural appropriateness, four experts (66.67%) agreed that the module considers the diverse cultural, religious, and socioeconomic backgrounds of adolescents in Sri Lanka. Nevertheless, the consultant psychiatrists highlighted that these aspects are not specifically tailored to adolescent populations.

Lower levels of agreement were observed for items related to the cultural sensitivity of examples, scenarios, and language used, as well as their appropriateness for Sri Lankan adolescents, with three experts (50%) expressing agreement. The consultant psychiatrist indicated that although these are not specifically adolescent-focused, they are generally applicable within the Sri Lankan context and recommended further simplification of the language.

Additionally, three experts (50%) agreed that the activities and exercises are engaging and developmentally appropriate. The consultant psychiatrist questioned whether these are more developmentally appropriate for parents than for adolescents, while the consultant community physician indicated that further explanations are needed. Finally, three experts (50%) agreed that the module addresses the pressures related to academic and familial expectations, with the consultant psychiatrist noting that these issues are reasonably addressed through selected case scenarios, stories, and discussion.

Agreement among experts on assessment items pertaining to clinical and public health perspectives was assessed by consultant community physicians and consultant psychiatrists. Three experts (75%) agreed that the coping strategies and interventions presented are effective and feasible within the Sri Lankan context. Qualitative feedback from the consultant psychiatrist supported the practical and contextual relevance of the strategies used in the module; however, it was emphasized that their effectiveness should be confirmed through implementation and outcome evaluation.

Experts’ agreement on assessment items for clinical and public health perspectives

Regarding whether the module addresses the most pressing mental health concerns among adolescents in Sri Lanka, two experts (50%) agreed. Qualitative feedback from the consultant psychiatrists indicated that the module does not directly address clinical mental health conditions but instead functions as a preventive, parent-focused mental health promotion tool. Similarly, two experts (50%) agreed that the mental health disorders and symptoms are described in a culturally understandable and non-stigmatizing manner within the Sri Lankan context. However, the consultant psychiatrist disagreed with this statement, noting that the module content predominantly addressed emotional and behavioral issues or disturbances rather than clinically defined mental health disorders (Table 2).

**Table 2:** Overall agreement on subject-specific assessment items by consultant psychiatrists and community physicians (n=4)

No.	Assessment items (statements)	Agreement - n (%)
1	The module addresses the most pressing mental health concerns among adolescents in Sri Lanka. (Statement_9)	2 (50)
2	The mental health disorders and symptoms are described in a way that is culturally understandable and not stigmatizing within the Sri Lankan context. (Statement_10)	2 (50)
3	The coping strategies and interventions presented are effective and feasible within the Sri Lankan context. (Statement_11)	3 (75)

Subject-specific assessment of Manohari module content by consultant psychiatrist (n = 2)

**Figure 1:**
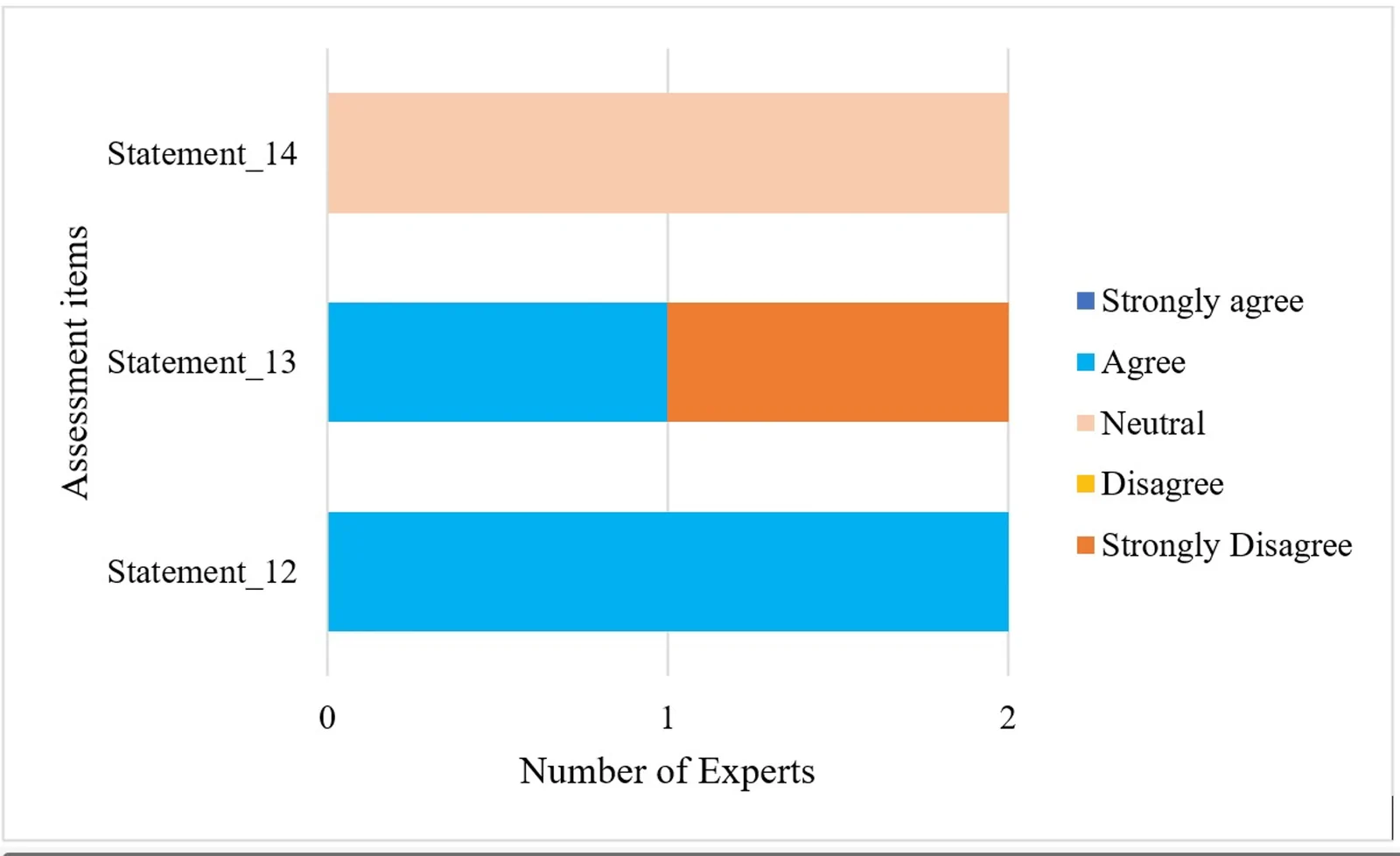
Ratings of the Manohari module content across subject-specific assessment items by consultant psychiatrists

Both psychiatrists (100%) agreed that the module adequately addresses issues such as stigma, help-seeking, and access to mental health services. However, qualitative feedback revealed nuanced differences: one psychiatrist acknowledged that these issues are adequately covered, whereas the other noted that access to mental health services is not sufficiently discussed, suggesting variability in the perceived comprehensiveness of the module (Figure 1).

In contrast, opinions diverged regarding the clinical soundness of the module from the perspective of mental health professionals (psychiatrists, psychologists, and counselors) in Sri Lanka. One psychiatrist (50%) agreed that the module is clinically sound, while the other (50%) strongly disagreed. The dissenting view emphasized that the module is better conceptualized as a mental health promotion or prevention tool rather than a clinically oriented intervention for treatment.

Regarding alignment with the current Sri Lankan national mental health policies, both psychiatrists (100%) provided neutral responses. Qualitative comments indicated uncertainty in this domain; one psychiatrist suggested that while the module may contribute to mental health promotion, it does not demonstrate clear alignment with broader adolescent mental health aspects.

Subject-specific assessment of Manohari module content by ZDE (n = 2)

**Figure 2:**
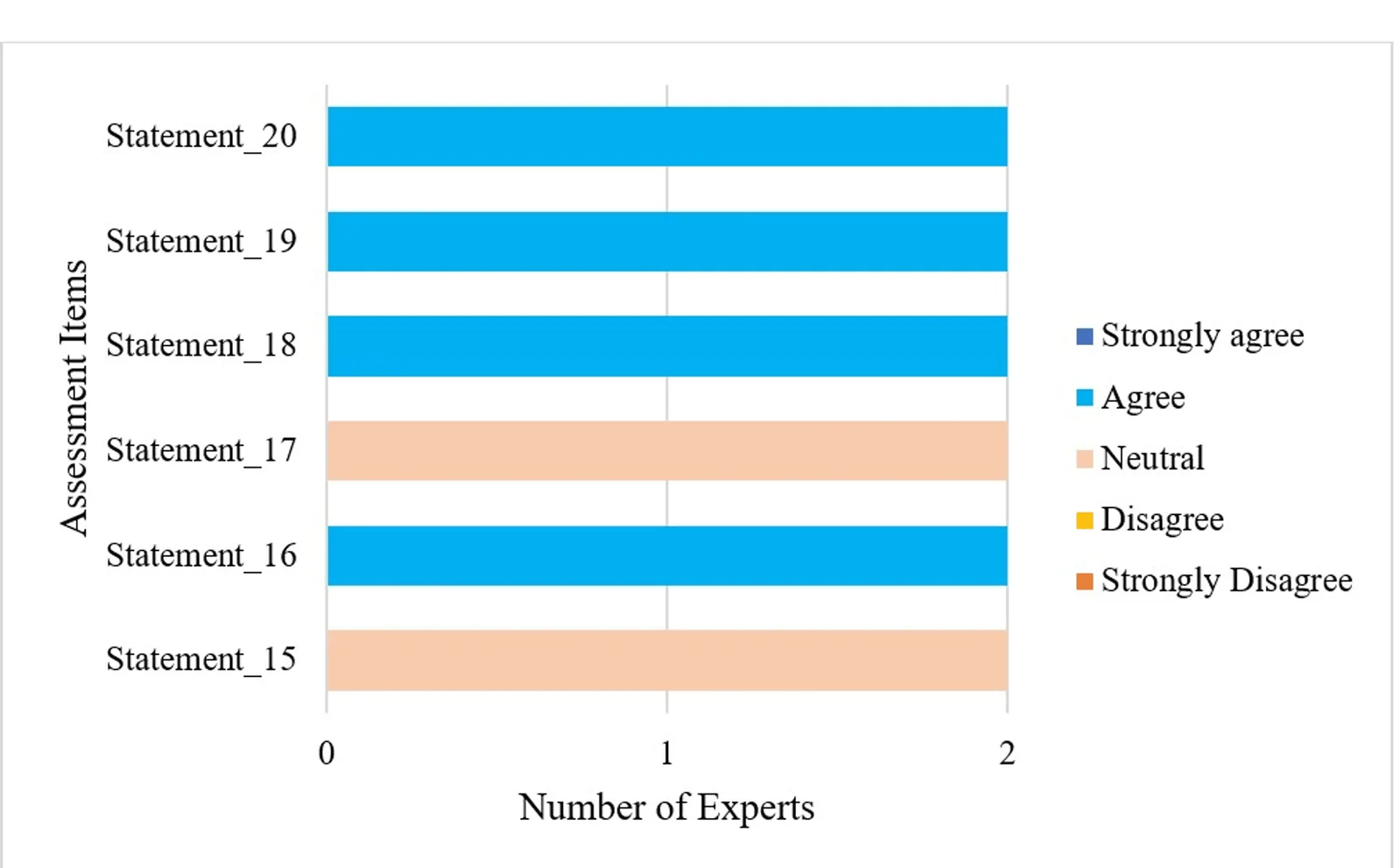
Ratings of the Manohari module content across subject-specific assessment items by Zonal Directors of Education (ZDE)

The responses to the subject-specific assessment items of the ZDEs indicate a high level of consensus across several domains, with some areas of neutrality (Figure 2).

Both ZDEs (100%) agreed that the visuals and supplementary materials used in the module are helpful and easy to understand. Similarly, both experts (100%) agreed that the module can help families support their adolescents' mental health and that teachers and school counsellors in Sri Lanka would find the module practical and applicable in educational settings. In addition, both ZDEs (100%) agreed that the module is compatible with the existing school curriculum and available resources, indicating strong alignment with the educational context.

However, both ZDEs (100%) provided neutral responses regarding whether community leaders and family members would find the module helpful and culturally appropriate, as well as whether the language and complexity of the content are suitable for the cognitive development of adolescents. This neutrality indicates a level of uncertainty and highlights the need for further contextual validation in these areas.

Qualitative feedback from the experts

Qualitative feedback from the experts provided additional depth to the judgmental validation findings, highlighting both strengths and areas requiring refinement.

The psychiatrist identified a few adolescent-specific issues that need more attention within the module, including predominant reliance on knowledge-based content rather than skill-based development, limited coverage of digital challenges and cyber risks, and insufficient exploration of the generation gap between traditional beliefs and modern adolescent aspirations. The inclusion of components focusing on resilience and empowerment was also recommended. Despite these concerns, the psychiatrist acknowledged that the modules are innovative, practical, and culturally aligned. However, it was noted that the translation of these perceived benefits into measurable outcomes remains uncertain, given the potential influence of multiple factors on implementation.

Community physicians highlighted several practical and content-related limitations. It was noted that exercises could be explained more clearly, with greater emphasis on key follow-up issues such as parental attitudes (e.g., comparing children with others) and the prevention of adolescent substance use. Additionally, concerns were raised regarding the limited coverage of mental health disorders and symptoms, with recommendations to include these aspects to enhance the comprehensiveness of the module. Further feedback emphasized the need to better incorporate context-specific challenges faced by adolescents in Sri Lanka. In particular, the significant pressure arising from high parental expectations regarding academic achievement was identified as a major contributor to sustained stress among adolescents.

Moreover, the community physician highlighted important issues such as the relatively high rates of adolescent suicide in the Northern Province, often linked to academic pressure from parents and teachers, interpersonal conflicts, and relationship-related stresses. Road traffic injuries and fatalities among late adolescents were identified as a neglected area. In addition, the importance of addressing adolescents' understanding of sexual and emotional experiences, as well as supporting informed decision-making while balancing academic demands, was emphasized.

FGD findings

The thematic analysis of the FGD identified eight major themes related to the adolescents' perceptions of the Manohari module: (i) overall appeal of the module, (ii) relatability of characters and situations, (iii) understanding and application of life skills, (iv) language and clarity, (v) cultural appropriateness, (vi) portrayal of mental health, (vii) illustrations and visual appeal, and (viii) peer acceptability and recommendations.

Overall, participants expressed positive impressions of the module, describing the stories as interesting, engaging, and enjoyable to read. Several students highlighted that the variety of situations presented helped maintain their attention. However, some suggested that certain story titles could be improved to better reflect the content and recommended incorporating more humor to enhance engagement and age-appropriateness.

In terms of relatability of characters and situations, participants agreed that the characters and situations were realistic and reflective of their daily experiences. They noted that the problems depicted were similar to those encountered in their own lives. However, students preferred the use of real-life human characters (e.g., parents, siblings, and friends) rather than animals, reflecting a preference for real-life relevance.

Participants demonstrated a strong understanding of the life skills embedded in the stories, particularly in areas such as decision-making, emotional regulation, and communication. They reported that these concepts were clearly conveyed through the narratives. Furthermore, most students agreed that the life skills presented were applicable to real-life situations. When asked to rate the influence of the stories on their understanding or behavior (on a scale of 1-10), the majority rated it as 6/10, suggesting a moderate impact, which they attributed to insufficient motivational elements within the module.

Regarding language and clarity, students consistently reported that the content was simple, clear, and easy to understand, with minimal use of complex vocabulary. This enabled them to complete the reading within a relatively short time (approximately 30 minutes).

The module was also perceived as culturally appropriate, with participants noting that the family dynamics and peer interactions reflected typical Sri Lankan contexts. However, some students pointed out that certain character names were not culturally suitable, suggesting the need for minor adjustments.

In relation to the portrayal of mental health, participants responded positively, indicating that emotional experiences were presented in a non-judgmental and normalized manner. They suggested that incorporating more humor and explanatory elements could further enhance emotional engagement and potentially contribute to stress relief and improved emotional well-being.

Feedback on illustrations was generally favorable, with students noting that visuals supported their understanding of the stories. However, they recommended including more illustrations, particularly cartoon-style images with humorous elements, to increase engagement.

Finally, there was strong consensus regarding the module’s peer appeal and acceptability. Participants indicated that the stories were relatable and did not feel like additional academic work. All students expressed willingness to recommend the module to their peers, suggesting high acceptability within the target group.

Overall, the FGD findings indicate that the module is engaging, culturally relevant, and understandable, with opportunities for improvement in areas such as motivation, humor, character representation, and contextual refinement.

## Discussion

The study assessed the content validity and cultural relevance of the Manohari module to support its implementation among adolescents in Sri Lanka using a mixed-method approach. Overall, the findings indicate that the module is acceptable and culturally relevant while highlighting several areas requiring refinement to improve its effectiveness and applicability.

The positive expert appraisal of the module’s content, cultural appropriateness, and practicality for delivery by non-health professionals supports its potential as a scalable intervention. This is consistent with established evidence that life skills-based mental health promotion interventions can be effectively implemented in low- and middle-income settings when delivered by trained lay facilitators, thereby increasing scalability and accessibility [14,25].

However, the qualitative feedback highlights several critical areas for refinement. Notably, concerns were raised regarding the developmental appropriateness of the content, particularly its limited relevance to late adolescence. This is consistent with existing literature indicating that adolescence comprises distinct developmental stages, including early, middle, and late adolescence, each characterized by differences in cognitive, emotional, and social functioning [26], thereby underscoring the importance of interventions that are appropriately tailored to these developmental variations. Overall, while the module demonstrates strong foundational potential as a practical tool for school-level mental health promotion, further refinement in terms of developmental targeting is essential to enhance its relevance, acceptability, and effectiveness among Sri Lankan adolescents.

A notable finding of this study was the lack of consensus between the two psychiatrists regarding the clinical soundness of the Manohari module. While one psychiatrist considered the module to be clinically sound, the other strongly disagreed, indicating that consensus was not achieved on an important aspect of the module. This difference in expert opinion highlights the need for continued refinement and further validation of the module by a larger multidisciplinary expert panel.

The variation in expert agreement regarding clinical relevance also underscores the importance of clearly positioning the module within a preventive and promotive framework. As indicated by the experts, the module’s primary focus on emotional and behavioral competencies, rather than clinically defined mental health disorders, aligns with existing literature indicating that adolescent health interventions are intersectoral and multi-component, incorporating school- and community-based approaches and are associated with improvements in psychosocial functioning and well-being [2,23,27]. This suggests that while the module is appropriate as a mental health promotion resource, its role should be clearly positioned within a preventive framework and not as a substitute for clinical mental health care.

From an implementation perspective, the views of ZDEs regarding the module’s practicality, clarity of visuals, and compatibility with the existing school curriculum suggest strong potential for integration within the educational system. However, the neutrality expressed regarding cultural appropriateness for broader community stakeholders highlights the need for further contextual validation.

The findings from the FGDs indicate that the module is highly acceptable, engaging, and understandable from the perspective of adolescents, supporting its potential as a youth-friendly mental health promotion tool. The positive reception of the stories, particularly their relatability and alignment with real-life experiences, highlights the importance of interventions being acceptable and perceived as relevant by participants in order to promote engagement [28]. Participants’ preferences for realistic human characters over symbolic representations further highlight the importance of authenticity and contextual relevance in adolescent-focused interventions. The strong understanding of life skills such as decision-making, emotional regulation, and communication suggests that the module effectively conveys key competencies, aligning with the principles of life skills education promoted by WHO, which emphasize experiential and relatable learning approaches. However, the moderate self-reported impact (mean rating 6/10) and suggestions for increased humor, motivation, and illustrative content indicate that while the module is informative, its capacity to influence behavior change may be limited without stronger engagement strategies. Overall, these findings suggest that while the module is well-designed in terms of content clarity, cultural alignment, and acceptability, incorporating more engaging and motivational components could enhance its effectiveness in promoting meaningful behavioral and emotional change among adolescents.

While the above findings highlight the relevance and acceptability of the Manohari module within the Sri Lankan context, several limitations should be considered when interpreting the results. A key limitation of this study is the relatively small number of participants included in both the expert panel and the FGD, which may limit the generalizability of the findings. Although participants were selected based on their expertise and involvement in adolescent mental health, the sample may not fully represent the diversity of perspectives across different regions and professional groups in Sri Lanka.

In addition, the study relied primarily on subjective perceptions and self-reported feedback to evaluate the module. Such approaches are inherently susceptible to response bias, which may have influenced the assessment outcomes. Moreover, inter-rater reliability was not formally assessed using statistical measures such as Cohen’s kappa, Fleiss’ kappa, or the intraclass correlation coefficient (ICC). Although the level of agreement among experts was reported using raw agreement percentages, this approach does not account for agreement occurring by chance and provides a less robust assessment of consensus compared with established reliability statistics.

Furthermore, a potential conflict of interest may exist due to the involvement of the developer of the instrument as a co-author of the study. This dual role may have introduced the possibility of bias during the evaluation or interpretation of findings. Although efforts were made to maintain objectivity through expert review and systematic evaluation procedures, future studies should consider involving independent researchers without direct involvement in the process to enhance transparency and reduce potential bias. Beyond this, the study did not incorporate objective measures to assess the effectiveness of the module, such as pre- and post-intervention evaluations among adolescents. As a result, the findings are limited to perceived relevance and acceptability rather than demonstrable impact. Future research involving larger and more diverse samples, as well as the inclusion of objective outcome measures, would be valuable in strengthening the evidence base and supporting wider implementation of the module.

## Conclusions

This study provides preliminary evidence supporting the acceptability, content validity, and cultural relevance of the Manohari module as a life skills development package for adolescents in Sri Lanka. The positive evaluations from experts and adolescents highlight its potential for implementation as a school-based intervention. However, several areas for refinement were identified, including the need for improved developmental targeting and greater contextual adaptation to address adolescent-specific challenges. Furthermore, the module should be clearly positioned as a preventive and promotive tool rather than a component of clinical mental health care.

Future research should focus on validating the Manohari module with larger and more diverse panels of experts and adolescent participants, followed by pilot implementation and outcome evaluation to assess its effectiveness in improving adolescent mental health and life skills, as well as to inform its integration into existing educational and public health systems.

## Appendices

Appendix 1

**Table 3:** Evaluation form for psychiatrist

No	Assessment items (Statements)	Strongly agree	Agree	Neutral	Disagree	Strongly disagree	Remarks
1	The topics covered are relevant to the adolescent population. (Statement_1)						
2	The module aligns with the current understanding of adolescent mental health in Sri Lanka. (Statement_2)						
3	The learning activities are practical and easily understandable for non-health professionals delivering the module. (Statement_3)						
4	The module adequately covers key life skills needed for the mental health promotion. (Statement_4)						
5	The examples, scenarios, and language used are culturally sensitive and appropriate for Sri Lankan adolescents. (Statement_5)						
6	The module considers the diverse cultural, religious, and socioeconomic backgrounds of adolescent in Sri Lanka. (Statement_6)						
7	The activities and exercises are engaging and developmentally appropriate. (Statement_7)						
8	The module addresses the pressure from academic expectations and familial expectations. (Statement_8)						
9	The module addresses the most pressing mental health concerns among adolescents in Sri Lanka (Statement_9)						
10	The mental health disorders and symptoms described in a way that is culturally understandable and not stigmatizing within Sri Lankan context. (Statement_10)						
11	The coping strategies and interventions presented are effective and feasible within Sri Lankan context. (Statement_11)						
12	The module adequately addresses issues like stigma, help-seeking, and access to mental health services. (Statement_12)						
13	The mental health professionals (psychiatrists, psychologists, and counselors) in Sri Lanka consider the module to be clinically sound. (Statement_13)						
14	The module aligns with the current Sri Lankan national mental health policies. (Statement_14)						
15	Are there any important topics or issues that are missing from the module?						
16	Remarks						

**Table 4:** Evaluation form for community physician

No	Assessment items (Statements)	Strongly agree	Agree	Neutral	Disagree	Strongly disagree	Remarks
1	The topics covered are relevant to the adolescent population. (Statement_1)						
2	The module aligns with the current understanding of adolescent mental health in Sri Lanka. (Statement_2)						
3	The learning activities are practical and easily understandable for non-health professionals delivering the module. (Statement_3)						
4	The module adequately covers key life skills needed for the mental health promotion. (Statement_4)						
5	The examples, scenarios, and language used are culturally sensitive and appropriate for Sri Lankan adolescents. (Statement_5)						
6	The module considers the diverse cultural, religious, and socioeconomic backgrounds of adolescent in Sri Lanka. (Statement_6)						
7	The activities and exercises are engaging and developmentally appropriate. (Statement_7)						
8	The module addresses the pressure from academic expectations and familial expectations. (Statement_8)						
9	The module addresses the most pressing mental health concerns among adolescents in Sri Lanka (Statement_9)						
10	The mental health disorders and symptoms described in a way that is culturally understandable and not stigmatizing within Sri Lankan context. (Statement_10)						
11	The coping strategies and interventions presented are effective and feasible within Sri Lankan context. (Statement_11)						
12	Community leaders and family members will find the module helpful and culturally appropriate. (Statement_15)						
13	Are there any important topics or issues that are missing from the module?						
14	Remarks						

**Table 5:** Evaluation form for ZDE

No	Assessment items (Statements)	Strongly agree	Agree	Neutral	Disagree	Strongly disagree	Remarks
1	The topics covered are relevant to the adolescent population. (Statement_1)						
2	The module aligns with the current understanding of adolescent mental health in Sri Lanka. (Statement_2)						
3	The learning activities are practical and easily understandable for non-health professionals delivering the module. (Statement_3)						
4	The module adequately covers key life skills needed for the mental health promotion. (Statement_4)						
5	The examples, scenarios, and language used are culturally sensitive and appropriate for Sri Lankan adolescents. (Statement_5)						
6	The module considers the diverse cultural, religious, and socioeconomic backgrounds of adolescent in Sri Lanka. (Statement_6)						
7	The activities and exercises are engaging and developmentally appropriate. (Statement_7)						
8	The module addresses the pressure from academic expectations and familial expectations. (Statement_8)						
9	Community leaders and family members will find the module helpful and culturally appropriate. (Statement_15)						
10	The visuals and other materials used in the module are helpful and easy to understand. (Statement_16)						
11	The language and complexity of the content are suitable for the cognitive development of the adolescents. (Statement_17)						
12	The module provides information that can help families support their adolescents’ mental health. (Statement_18)						
13	The teachers and school counsellors in Sri Lanka will find the module useful and practical for use in educational settings. (Statement_19)						
14	The module is compatible with the existing school curriculum and resources. (Statement_20)						
15	Are there any important topics or issues that are missing from the module?						
16	Remarks						
